# Genome Sequences of Two Multidrug-Resistant *Proteus mirabilis* Strains Harboring CTX-M-65 Isolated from Malaysia

**DOI:** 10.1128/genomeA.01301-16

**Published:** 2016-11-17

**Authors:** Choo Yee Yu, Geik Yong Ang, Yun Fong Ngeow, Kok Keng Tee, Wai-Fong Yin, Kok-Gan Chan

**Affiliations:** a Division of Genetics and Molecular Biology, Institute of Biological Sciences, University of Malaya, Kuala Lumpur, Malaysia; b Integrative Pharmacogenomics Institute (iPROMISE), Universiti Teknologi MARA, Kuala Lumpur, Malaysia; c Department of Pre-Clinical Sciences, Faculty of Medicine and Health Sciences, Universiti Tunku Abdul Rahman, Kajang, Malaysia; d Department of Medical Microbiology, Faculty of Medicine, University of Malaya, Kuala Lumpur, Malaysia

## Abstract

*Proteus mirabilis* is an opportunistic nosocomial pathogen that is commonly associated with urinary tract infections. Here, we present draft genome sequences of two multidrug-resistant *P. mirabilis* strains, isolated from urine samples in Malaysia, that harbored a CTX-M-type extended-spectrum β-lactamase-encoding gene, as well as a repertoire of other antimicrobial-resistant determinants.

## GENOME ANNOUNCEMENT

*Proteus mirabilis* is a ubiquitous Gram-negative bacterium that can thrive in various settings such as polluted water, soils, and animals (1). From the clinical perspective, *P. mirabilis* is an important causative agent of complicated urinary tract infections and is also implicated in a range of other nosocomial infections such as bacteremia, empyema, and osteomyelitis (2). In recent years, the emergence of extended-spectrum β-lactamase (ESBL)-producing *P. mirabilis* strains has become a growing concern, as they are associated with poor treatment outcome and prolonged hospitalization (2–4).

In the present study, two *P. mirabilis* strains, designated PM_125 and PM_178, were recovered from patient urine specimens in 2013. The isolates were identified using the Vitek 2 system (bioMérieux, Marcy l’Etoile, France) and matrix-assisted laser desorption/ionization time-of-flight mass spectrometry (Bruker Daltonik GmbH, Bremen, Germany). Antibiotic susceptibility tests revealed that both strains tested positive for ESBL and were resistant to ampicillin, ampicillin-sulbactam, piperacillin, ticarcillin, cefazolin, cephalothin, cefuroxime, ceftriaxone, cefpodoxime, cefotaxime, doripenem, nalidixic acid, moxifloxacin, tetracycline, nitrofurantoin, and trimethoprim-sulfamethoxazole, while remaining susceptible to cefoxitin, ceftazidime, imipenem, aztreonam, and norfloxacin when interpreted according to EUCAST Breakpoints version 6 (http://www.eucast.org/clinical_breakpoints).

Genomic DNA of PM_125 and PM_178 was extracted using the Epicenter MasterPure DNA purification kit (Madison, WI, USA), and whole-genome sequencing was performed on an Illumina HiSeq 2000 platform (San Diego, CA, USA). *De-novo* assembly of the raw reads was conducted using CLC Genomics Workbench version 7.0 (Aarhus, Denmark), and the genomes were annotated using the NCBI Prokaryotic Genome Annotation Pipeline version 2.10 and Rapid Annotations using Subsystem Technology server version 2.0 (5), while the antimicrobial-resistant genes were identified using ResFinder (https://cge.cbs.dtu.dk/services/ResFinder). A total of 149 and 190 contigs were obtained for PM_125 and PM_178, respectively. The genome of PM_125 was 3,955,474 bp in size (38.8% G+C content) and a total of 3,469 protein-coding sequences were predicted, whereas the 3,969,065-bp genome (38.9% G+C content) of PM_178 was predicted to contain 3,475 protein-coding sequences. The draft genomes of PM_125 and PM_178 shared an average nucleotide identity of 100%.

Various antimicrobial resistance genes were identified in both genomes, including genes conferring resistance to aminoglycosides [*aadA1*, *aac(6′)Ib-cr*, *aac(3)-IVa*, *aph(4)-Ia*, *strA*, *strB*], β-lactams (*bla*_CTX-M-65_, *bla*_OXA-1_), fluoroquinolone [*aac(6′)Ib-cr*], fosfomycin (*fosA*), phenicols (*floR, catB3*), rifampin (*arr-3*), sulfonamide (*sul1*, *sul2*, *sul3*), tetracycline [*tet(A)*, *tet(J)*], and trimethoprim (*dfrA1*). In addition to this, three types of resistance modules were found: (i) the *bla*_CTX-M-65_ bracketed by two IS*26* elements; (ii) an array of *aac(6′)Ib*-*bla*_OXA-1_-*catB3*-*arr-3-∆qacE*-*sul1*; and (iii) an array of *strA*-*strB*-*orf*_unknown_-*tetR*-*tetA*. The presence of multidrug efflux pump–mediated resistance mechanisms was also inferred from the genomes.

In conclusion, we present the draft genomes of two multidrug-resistant *P. mirabilis* strains from Malaysia, which, to the best of our knowledge, also represent the first report of CTX-M-65-producing *P. mirabilis* genomes. These genomes offer an avenue for further investigations into antimicrobial-resistance determinants in ESBL-positive *P. mirabilis* strains and also facilitate basic biological research by allowing data to be mined from the genetic information that have been made publicly available.

### Accession number(s).

The whole-genome shotgun projects of *P. mirabilis* PM_125 and PM_178 have been deposited in DDBJ/ENA/GenBank under the accession numbers LWUL00000000 and LWUM00000000, respectively.

## References

[1] O’Hara CM, Brenner FW, Miller JM 2000 Classification, identification, and clinical significance of *Proteus*, *Providencia*, and *Morganella*. Clin Microbiol Rev 13:534–546. doi:10.1128/CMR.13.4.534-546.2000. 11023955 PMC88947

[2] Chen CY, Chen YH, Lu PL, Lin WR, Chen TC, Lin CY 2012 *Proteus mirabilis* urinary tract infection and bacteremia: risk factors, clinical presentation, and outcomes. J Microbiol Immunol Infect 45:228–236. doi:10.1016/j.jmii.2011.11.007. 22572004

[3] Tumbarello M, Trecarichi EM, Fiori B, Losito AR, D’Inzeo T, Campana L, Ruggeri A, Di Meco E, Liberto E, Fadda G, Cauda R, Spanu T 2012 Multidrug-resistant *Proteus mirabilis* bloodstream infections: risk factors and outcomes. Antimicrob Agents Chemother 56:3224–3231. doi:10.1128/AAC.05966-11. 22450979 PMC3370815

[4] Endimiani A, Luzzaro F, Brigante G, Perilli M, Lombardi G, Amicosante G, Rossolini GM, Toniolo A 2005 *Proteus mirabilis* bloodstream infections: risk factors and treatment outcome related to the expression of extended-spectrum beta-lactamases. Antimicrob Agents Chemother 49:2598–2605. doi:10.1128/AAC.49.7.2598-2605.2005. 15980325 PMC1168714

[5] Aziz RK, Bartels D, Best AA, DeJongh M, Disz T, Edwards RA, Formsma K, Gerdes S, Glass EM, Kubal M, Meyer F, Olsen GJ, Olson R, Osterman AL, Overbeek RA, McNeil LK, Paarmann D, Paczian T, Parrello B, Pusch GD, Reich C, Stevens R, Vassieva O, Vonstein V, Wilke A, Zagnitko O 2008 The RAST server: rapid annotations using subsystems technology. BMC Genomics 9:75. doi:10.1186/1471-2164-9-75. 18261238 PMC2265698

